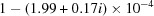# *Condor*: a simulation tool for flash X-ray imaging^1^

**DOI:** 10.1107/S1600576716009213

**Published:** 2016-07-14

**Authors:** Max F. Hantke, Tomas Ekeberg, Filipe R. N. C. Maia

**Affiliations:** a Laboratory of Molecular Biophysics, Department of Cell and Molecular Biology, Uppsala University, Husargatan 3 (Box 596), SE-751 24 Uppsala, Sweden; b NERSC, Lawrence Berkeley National Laboratory, Berkeley, California 94720, USA

**Keywords:** femtosecond coherent diffractive imaging, X-ray free-electron lasers, simulation, single-particle imaging, computer programs

## Abstract

Flash X-ray imaging has the potential to determine structures down to molecular resolution without the need for crystallization. The ability to accurately predict the diffraction signal and to identify the optimal experimental configuration within the limits of the instrument is important for successful data collection. This article introduces *Condor*, an open-source simulation tool to predict X-ray far-field scattering amplitudes of isolated particles for customized experimental designs and samples, which the user defines by an atomic or a refractive index model. The software enables researchers to test whether their envisaged imaging experiment is feasible, and to optimize critical parameters for reaching the best possible result. It also aims to support researchers who intend to create or advance reconstruction algorithms by simulating realistic test data. *Condor* is designed to be easy to use and can be either installed as a Python package or used from its web interface (http://lmb.icm.uu.se/condor). X-ray free-electron lasers have high running costs and beam time at these facilities is precious. Data quality can be substantially improved by using simulations to guide the experimental design and simplify data analysis.

## Abstract 2

*Condor*, an open-source simulation tool to predict X-ray scattering amplitudes for flash X-ray imaging experiments, is introduced.

## 1. Introduction   

Flash X-ray imaging (FXI) may become a tool to solve structures down to molecular resolution without the need for crystallization (Neutze *et al.*, 2000 ▸; Bergh *et al.*, 2008 ▸). By employing femtosecond pulses produced by X-ray free-electron lasers, FXI can outrun radiation damage processes that limit resolution (Chapman *et al.*, 2006 ▸). FXI dispenses with image forming lenses and thereby circumvents the difficulty of manufacturing efficient lenses for X-rays (Chapman & Nugent, 2010 ▸). Aersosol sample delivery avoids a sample support, which means that the structure can be imaged with practically no background (Bogan *et al.*, 2008 ▸; Seibert *et al.*, 2011 ▸; Hantke *et al.*, 2014 ▸).

For reaching the goal of 3 Å resolution, the Single Particle Imaging Initiative identifies the requirement of simulations that realistically represent the experiment conditions to guide future development (Aquila *et al.*, 2015 ▸). It is essential to optimize and harmonize all relevant experimental parameters, such as photon wavelength, photon flux, illumination profile, camera distance, detector settings, sample density and even sample type. Being able to accurately predict diffraction data facilitates optimization of the experimental setup and helps to provide accurate estimates of the expected data quality. Simulation tools can help researchers to use their beam time more efficiently and measure diffraction data at the highest possible quality.

Software for simulating X-ray diffraction data exists. For crystal diffraction, for example, *CCP4* (Winn *et al.*, 2011 ▸) is widely used. But it is aimed at crystal diffraction, making it hard to use for simulating continuous diffraction patterns. In a couple of publications (Yefanov & Vartanyants, 2013 ▸; Serkez *et al.*, 2013 ▸; Ayyer *et al.*, 2015 ▸) the program *Moltrans* is mentioned and described as a software package to simulate FXI data for atomic models. Unfortunately, the code is not openly available. Very recently, *SimS2E* was released, which is a very sophisticated start-to-end simulation framework specialized for single-molecule FXI at the European X-ray free-electron laser (Yoon *et al.*, 2015 ▸). A practical, convenient and openly available FXI software tool for a range of sample models is missing.

Here we introduce *Condor*, an easy-to-use software package to simulate FXI far-field scattering amplitudes from an experimental setup customized by the user. The user may define the sample either by atom positions or at lower resolution by a three-dimensional refractive index map. This allows one to simulate diffraction from samples that are unknown at atomic resolution but for which low-resolution densities from, for example, electron microscopy studies exist. Common challenges that a researcher faces with real data (Seibert *et al.*, 2011 ▸; Loh *et al.*, 2012 ▸; Hantke *et al.*, 2014 ▸; van der Schot *et al.*, 2015 ▸; Ekeberg *et al.*, 2015 ▸) can be introduced by adding, for example, noise, signal variation, missing data regions, fluctuation of the beam tilt, sample heterogeneity or sample contamination. So far, *Condor* has demonstrated its usefulness for the preparation of experiments, data validation (Hantke *et al.*, 2014 ▸), and the development of new software and algorithms (Daurer *et al.*, 2016 ▸).

**Figure 1:**
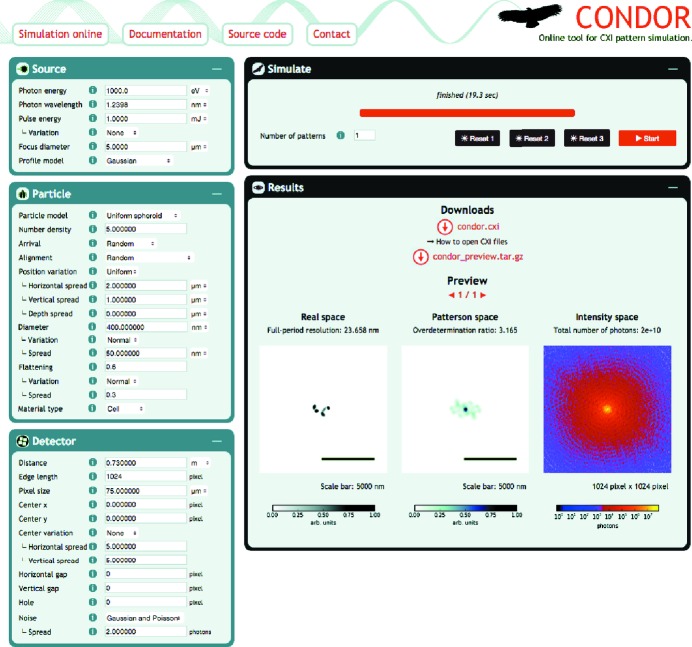
The interface of *Condor*’s web application (http://lmb.icm.uu.se/condor). In the left panels the user configures the source, particle and detector model. From the upper-right panel the job is submitted, and after the job has completed a preview and download links of the simulated data appear in the lower-right panel.

*Condor* is distributed under the free open-source Simplified Berkeley Software Distribution (BSD) License to ensure transparency and to ease future development and availability of the code. The source code can be downloaded from http://github.com/mhantke/condor. *Condor* does not require a local installation. It can be used directly from its web interface at http://lmb.icm.uu.se/condor (Fig. 1 ▸).

In this paper we give a description of the theoretical diffraction model that the code is based on (§[Sec sec2]2), describe how to use *Condor* (§3) and outline details of the current implementation (§4). The last chapter summarizes the paper and draws conclusions (§5).

## 2. Theory   

*Condor* attempts to predict coherent X-ray diffraction patterns on the basis of a sample model. Below we briefly outline the necessary approximations and the derivation of the scattering formulas that are used. For a comprehensive description of the theory behind, see, for example, Paganin (2006 ▸) and Als-Nielsen & McMorrow (2001 ▸).

For X-ray energies far from any absorption edges and well below the rest mass energy of an electron (511 keV) we may neglect Compton scattering. The samples that are considered here have a thickness of up to a few hundred nanometres and interact, because of their small size, only weakly with X-rays. This circumstance allows us to neglect the perturbation of the primary wave by the scattered wave within the sample. This approximation is well known as the first-order Born approximation.

Predictions suggest that femtosecond X-ray pulses can outrun radiation damage processes (Neutze *et al.*, 2000 ▸). Hence, in the simulations we model the sample by a scattering potential 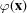, which is invariant over the duration of the pulse.

**Figure 2:**
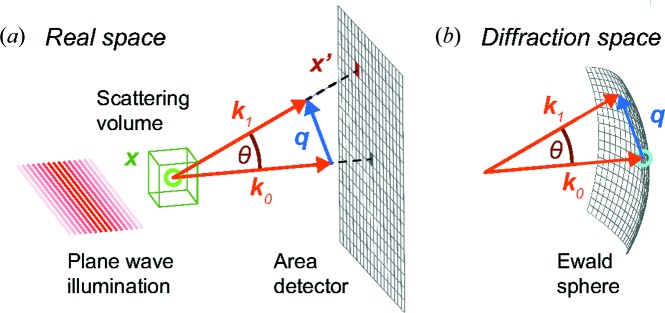
Schematic representation of the geometry in real and Fourier space. (*a*) A plane wave illuminates the sample. It is placed in vacuum and confined to the scattering volume illustrated by the green box. The signal at the detector plane is the superposition of the primary wave with wavevector 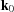 and the scattered wave with wavevector 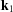. (*b*) The diffraction space is the reciprocal space of scattering vectors 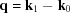 and contains the Fourier transform of the scattering potential 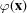.

The sample particle is placed in vacuum and illuminated by a plane wave with wavevector 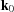 (see Fig. 2 ▸). We seek to predict the wavefield Ψ at pixel positions 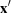 in the detector plane that is orthogonal to the beam axis and at a far distance from the object. In this scenario 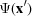 can be expressed as the sum of the primary wave 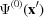 and the scattered wave (or scattering amplitude) 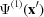. The direct beam 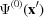 does not carry any structural information and is confined to the forward direction. It usually passes through a gap between the detector panels or is blocked by a beam stop and is never measured. Structural information about the sample is encoded by the scattering amplitude 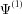, which is the superposition of spherical waves with amplitude 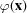 originating from all points 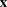 in the scattering volume:
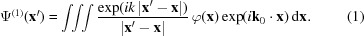
In our scenario, the sample volume is small and the detector distance large. Hence, we may safely assume 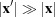 and obtain the far-field approximation of (1):
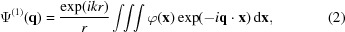
where 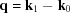 denotes the scattering vector and 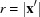. Since we only consider elastic scattering the energy is conserved and so is the wavenumber 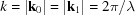, where λ denotes the wavelength. As we are only interested in relative phase differences we neglect the phase factor 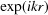 in the following equations.

For numerical calculation of the scattering amplitude 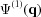 we have to either solve the integral in (2) or approximate it by a discrete function. Analytical solutions exist for certain sample models, such as uniformly filled spheres or spheroids (Feigin & Svergun, 1987 ▸; Hamzeh & Bragg, 1974 ▸). In *Condor* these solutions of (2) are implemented and can be customized by a few parameters. For more complex samples *Condor* provides two ways of defining the sample: either by a positional arrangement of atoms or by a gridded refractive index map. In the following subsections numerical solutions for these two particle models are presented. Both involve approximating the integral in (2) by discrete Fourier transforms (DFTs) that have the general form
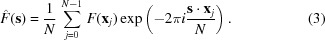
This formulation allows *Condor* to deploy efficient fast Fourier transform algorithms and exploit rapid parallel computing architectures.

### 2.1. Atomic model   

FXI studies often target small sample particles that have sufficient resemblance to systems for which atomic structures have been determined by either X-ray crystallography, cryo-electron microscopy or nuclear magnetic resonance spectroscopy. X-rays are scattered by atoms because of their bound electrons. The scattering strength of a single free electron is known as the Thomson scattering length 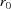. The scattering potential for *N* free electrons located at the respective positions 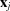 may be written as
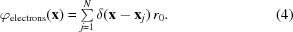
By substituting (4) into (2) the δ functions conveniently reduce the integral in (2) to a sum and we obtain the scattering amplitude in a simpler form:
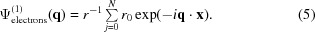



For electrons bound to an atom of species *a* the scattering length can be calculated by multiplying 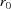 with the atomic scattering factor 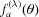. The atomic scattering factor is a semi-empirically determined element-specific constant that is tabulated for a large range of wavelengths λ and scattering angles θ (Brown *et al.*, 2006 ▸; Henke *et al.*, 1993 ▸). The shape of the atom is reflected in the angular dependency; hence the atomic scattering factor is also known as the atomic form factor.

This permits us to replace the integral in (2) as in (5) by a sum. The scattering amplitude can be evaluated by separating the calculation into sums for each atom species *a* that accounts for 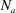 atoms at positions 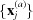. We obtain
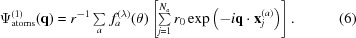




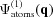
 now has the form of a sum of DFTs (3) with 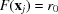 computed on the nonregular grid 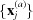.

### 2.2. Refractive index model   

For larger objects, such as big protein complexes or virus particles, the atomic structure is rarely on hand. However, at lower than atomic resolution electron density maps 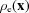 of a wide range of structures have been measured by electron microscopy. Also, for many relevant optical media we can estimate the atomic composition (see Table 1 ▸) and are able to model samples by customized density maps of optical media. For these cases the scattering potential 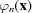 can be derived from the Maxwell equations and written as a function of the complex valued refractive index 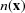:
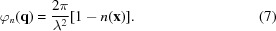
For convenience we define 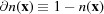. By inserting (7) into (2) we obtain the scattering amplitude as a function of 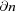:
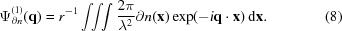
If this equation is interpreted as the continuum limit of (5) the relationship between the refractive index and the electron density distribution 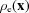 becomes
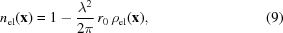
and the relationship between refractive index and the atom density distribution 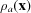 becomes
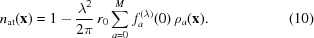
Using the relationships (9) and (10), *Condor* converts electron and atom density maps into refractive index maps. We presume here that 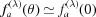 for all scattering angles θ, which is a valid assumption if the resolution of the measurement is well below atomic length scales.

Discretization of the Fourier integral in (2) with (3) on a three-dimensional cubic grid of 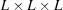 points at spacing 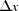 results in
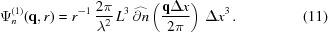
with 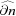 being the Fourier transform of 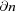. This expression allows *Condor* to efficiently calculate the scattering amplitude for any discrete map 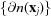 on the regular grid 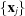.

### 2.3. Diffraction measurement   

To predict the absolute scattering signal 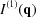 measured with a photon detector we need to take into account the intensity 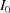 of the illumination, the solid angle 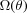 that is covered by the detector pixel, and the polarization factor 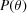, which accounts for the effects of the polarization of the incoming beam in the scattered signal (Als-Nielsen & McMorrow, 2001 ▸). With these parameters the expectation value for the number of scattered photons measured in a pixel (without noise and any losses) is given by
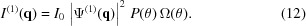



Owing to the quantum nature of photons the measurement of 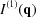 inevitably suffers from shot noise and thus follows Poisson statistics. This type and other types of measurement errors such as detector noise, parasitic scattering and limited quantum efficiency may be added to the simulated intensity values if desired.

**Figure 3:**
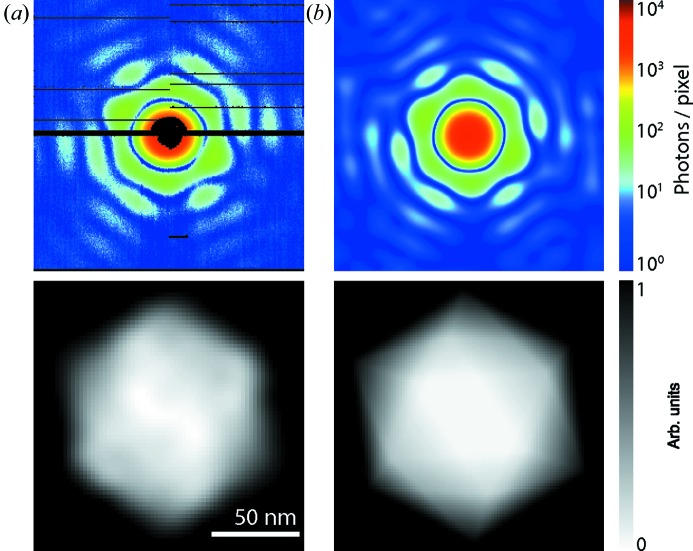
Comparison of experimental data and simulated data. (*a*) From the measured FXI diffraction pattern (top) of a single carboxysome (*i.e.* an icosahedral cell organelle) the projection image (bottom) was reconstructed by iterative phase retrieval down to 18.1 nm resolution. (*b*) At the given resolution the *Condor* simulation of a diffraction pattern and projection image for a uniformly filled icosahedron in matching orientation and size provides an acceptable approximation for the data shown in (*a*). Figure adapted from Hantke *et al.* (2014 ▸).

For the refractive index model the agreement of data from a real FXI experiment and simulated data calculated by using the formalism that has been described here is demonstrated in Fig. 3 ▸. For the atomic model such a comparison cannot be made because we lack suitable experimental data at this point.

## 3. Usage   

In the following paragraphs we give an introduction to the usage and functionality of *Condor*. For a detailed description of all features please see *Condor*’s documentation at http://lmb.icm.uu.se/condor/documentation.

Every *Condor* simulation requires the configuration of at least three components: the X-ray source, at least one sample and a pixel array detector. The configuration of the X-ray source defines the photon wavelength and intensity at the interaction point. The model of the sample can be of different kinds, either an atomic model or a refractive index description. The atomic description requires knowledge about all atom positions and atom species in the scattering volume. For example the online Protein Data Bank (PDB; Berman *et al.*, 2000 ▸) is a resource that provides a wide range of structures at atomic resolution. The structure can be provided either by a list of coordinates and atomic numbers or by a PDB file or PDB ID code.

To define a refractive index map *Condor* accepts a three-dimensional array of data points on a cubic Cartesian grid or the geometrical parameters of a sphere or spheroid. The map values can be refractive indices, electron densities or atom densities. For the last two, formulas (9) or (10) are used for the conversion to refractive indices. *Condor* interfaces to the Electron Microscopy Databank (EMDB; Lawson *et al.*, 2011 ▸), from which density maps can be retrieved. The orientation of the particle is defined by an extrinsic rotation. The rotation can be defined by either a triple of Euler angles, a rotation matrix or a quaternion. Multiple particles at different positions in the beam can be simulated as well. The configuration of the pixel detector determines the position of all pixels in space with respect to the interaction point. The detector noise, the fluctuating beam tilt, the saturation level, a missing data mask *etc*. may also be specified.

**Figure 4:**
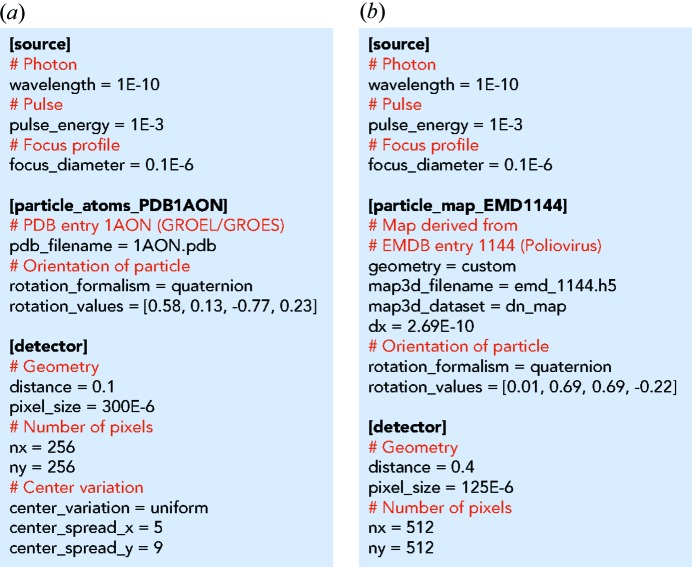
Configuration files for simulation with (*a*) an atomic and (*b*) a refractive index model. The source section defines the illumination properties, the particle section the sample properties and the detector section the parameters for the area pixel detector. Many parameters are optional and are set to default values if not specified. These configuration files together with the required structure files are included in the online repository of *Condor* and are located in the folder examples_publication.

The default way of carrying out a *Condor* simulation is by calling the executable condor from a folder that contains a configuration file named condor.conf. Fig. 4 ▸ shows two example configuration files, one for the calculation with an atomic model (Fig. 4 ▸ *a*) and one for the calculation with a refractive index model (Fig. 4 ▸ *b*). Every configuration file is subdivided into at least three sections [X-ray source, sample particle(s), pixel array detector]. All quantities follow the convention of the International System of Units. If a parameter is unspecified it is set to a default value. At the end of execution the results are written to an HDF5 file. The acronym HDF5 stands for Hierarchical Data Format version 5 (The HDF Group, 2016 ▸), which is a widely used file format for scientific applications and ensures high portability and performance. The data structure within the file follows the guidelines for the Coherent X-ray Imaging file format (Maia, 2012 ▸).

**Figure 5:**
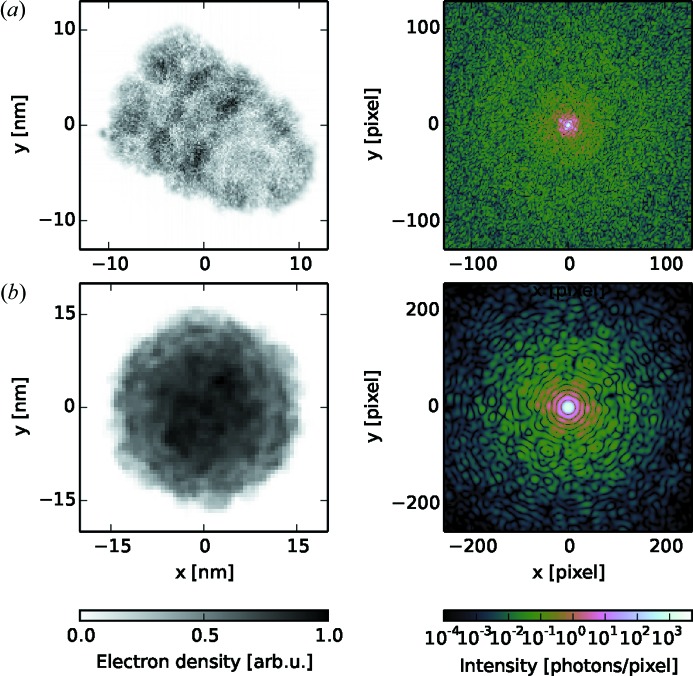
Simulation of diffraction patterns for two biological structures: (*a*) the GroEL–GroES complex and (*b*) the poliovirus particle. For each model the projection image is shown on the left and the noise-free intensity pattern on the right.

The two example configuration files shown in Fig. 4 ▸ define experimentally feasible configurations at the LINAC Coherent Light Source (LCLS). The selected particle structures are the GroEL–GroES protein complex (Fig. 4 ▸ *a*) and the poliovirus particle (Fig. 4 ▸ *b*). The structure for the GroEL–GroES protein complex is taken from the atom positions of PDB entry 1aon (Xu *et al.*, 1997 ▸). The poliovirus particle is modelled by the density map derived from EMDB entry 1144 (Bubeck *et al.*, 2005 ▸). We projected the EMDB map to electron densities using experimentally determined values for atomic composition (Molla *et al.*, 1991 ▸) and mass density (Dans *et al.*, 1966 ▸) of poliovirus virions. Simulated results from these examples are shown in Fig. 5 ▸.

*Condor* provides not only intensities but also phases. Here the curvature of the Ewald sphere is small, and hence projection images in real space (left column in Fig. 5 ▸) can be readily calculated by inverse Fourier transforming the scattering amplitudes.

**Figure 6:**
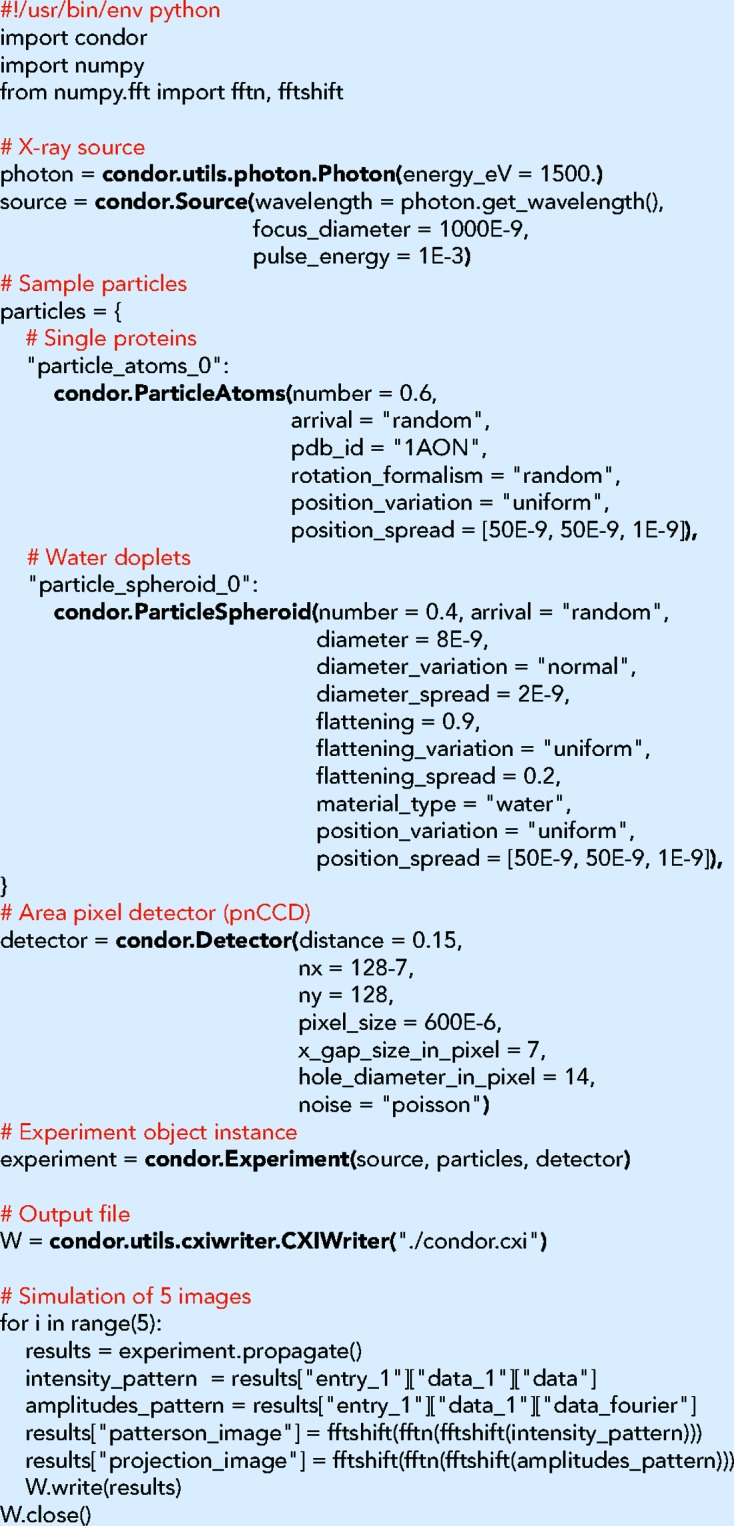
Customized use of *Condor* by direct interaction with the Python API. The script simulates diffraction patterns from a mixture of GroEL–GroES complex and spheroidal water droplets of varying shape and size. A missing data region and Poisson noise are taken into account for the intensity estimate at the detector pixels. After the initialization, patterns are simulated sequentially in a loop by calling the method propagate() and appending the results to an HDF5 file.

**Figure 7:**
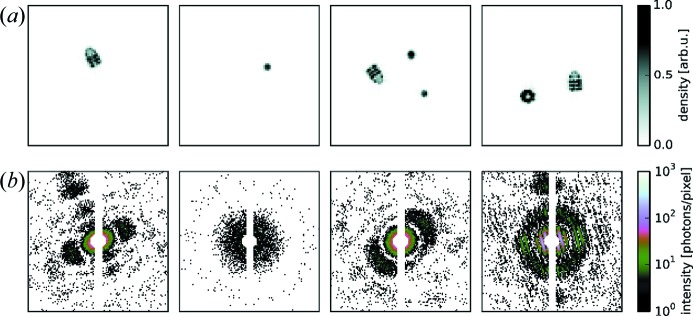
Simulation of diffraction patterns for a mixture of two particle species: the GroEL–GroES complex and spheroidal water droplets. (*a*) Real-space projection images and (*b*) respective simulated diffraction intensity patterns with Poisson noise and a pixel mask. The physical parameters resemble the conditions at the AMO beamline at the LCLS.

For a more customizable use, *Condor*’s application programming interface (API) can be called directly from any Python software. The *Condor* engine can thus be easily integrated into any software tool or pipeline that relies on simulated diffraction data. An example for a script that uses the *Condor* API is shown in Fig. 6 ▸. Projection images and diffraction patterns that were generated with this script are presented in Fig. 7 ▸. The script simulates an experiment where spheroidal water droplets contaminate the particle stream of GroEL–GroES protein complexes. Both particle species arrive in the scattering volume in random orientations and at random positions. The arrival statistics are modelled by a Poisson process with arrival rates of 0.2 for the water droplets and 0.9 for the protein complexes. The water droplets are not simulated as perfectly reproducible structures but as spheroids of varying size and shape. This is reflected in the model by size parameters that follow a normal distribution centred at 8 nm and values of the flattening parameter that follow a uniform distribution between 0.8 and 1.0.

## 4. Implementation   

*Condor* is a Python package including C extensions for the computationally heavy operations. For the calculation of the discrete Fourier transform in equations (6) and (11), *Condor* makes use of the non-equispaced fast Fourier transform (*NFFT*) C library (Keiner *et al.*, 2009 ▸). This library provides routines to calculate the discrete Fourier transform at non-equispaced points, for example on the curved surface of the Ewald sphere. For the refractive index model *Condor* deploys the common *NFFT* algorithm, which still requires equispaced sampling in the real-space domain. For the atomic model the generalized *NNFFT* algorithm is used, as it allows for non-equispaced sampling in both domains. The computation of the sums in the discrete Fourier transform can benefit from parallelization. Compilation with OpenMP (http://openmp.org) allows for an easy parallelization with moderate speed-ups. Diffraction from atomic models is normally more computationally demanding and here *Condor* supports the use of CUDA-capable graphics cards (http://nvidia.com/cuda), which can provide a drastic increase in performance.

Computation times were measured for the simulations of the examples shown in Figs. 4 ▸ and 5 ▸, which were carried out on a MacBookPro computer [2.5 GHz Intel Core i7 (4 cores, 8 threads), 16 GB 1600 MHz DDR3] equipped with a CUDA-capable graphics card (NVIDIA GeForce GT 750 M, 2048 MB memory). The atomic model included of 58 870 atom positions, and diffraction was predicted at 256 × 256 detector pixels. Using a single CPU and with CUDA disabled the calculation took 208 s. Enabling CUDA resulted in a computation time of 3 s, giving a speedup of 69.3×. The refractive index map consisted of 173 × 173 × 173 voxels, and diffraction was predicted at 512 × 512 detector pixels. Using a single CPU the calculation took 19 s, and using four CPU threads it took 6.8 s, resulting in a speed-up of 2.8×.

**Figure 8:**
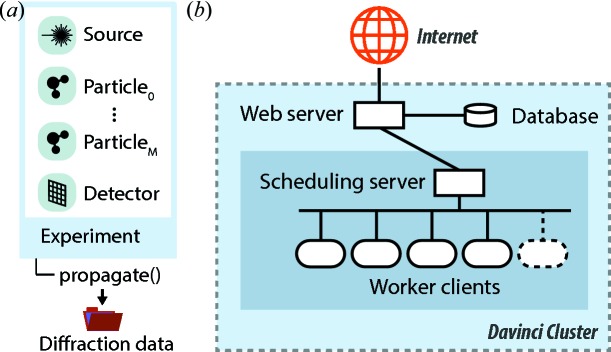
Implementation architecture. (*a*) An experiment is represented in *Condor* as a Python object that includes a source object, one or several sample particle objects, and a detector object. A call of the method propagate() starts a simulation and returns the diffraction data. (*b*) The web version of *Condor* is realized as a hierarchical client–server model. The web server provides a dynamic web page under the address http://lmb.icm.uu.se/condor. Under this page users can configure their experiment, and upon submission data are validated and then cached in a database. Simulation jobs are scheduled by a scheduling server that manages a network of worker clients. This worker farm is dynamically extended and shrunk depending on the number of requests. After completion of a simulation the web server presents previews and links to download to the user.

Fig. 8 ▸(*a*) illustrates the representation of an experiment in *Condor* as a Python object. It contains a source object, one or several particle objects, and a detector object. The experiment object has a method propagate() that starts the simulation of a single shot and returns the results in the form of a Python dictionary.

As an alternative to a local installation, *Condor* is also provided as a web application (Fig. 1 ▸) that supports most of the functionality of the full package. In the left panel of the web application one can configure the X-ray source, sample particle and detector. The upper right panel is used to submit simulation requests and monitor their progress. After a simulation has finished its results can be previewed and downloaded from the bottom right panel.

The web implementation of *Condor* is based on a Django (https://www.djangoproject.com/) web framework and uses a database for caching user inputs. The system is hosted by the Davinci GPU computer cluster of the Laboratory of Molecular Biophysics (Uppsala University, Sweden).

The architecture of the server–client model of the web implementation is illustrated in Fig. 8 ▸(*b*). When a user submits a simulation request the web server first checks the input. If the input passes validation the web server sends the requests to the *Condor* server, which manages a number of *Condor* clients. The first worker client that becomes available starts the *Condor* simulation. The number of worker clients is dynamically adjusted to the current load of the web page, such that at least one worker client is always available for processing a simulation request. The hierarchical architecture ensures responsiveness of the servers at all times, even when running multiple simulations simultaneously. While a simulation is running the scheduling server monitors the progress of the simulation. When finished the results are sent to the web server, which presents the user with previews and links for downloading the results as an HDF5 file.

## 5. Conclusion   

FXI experiments at free-electron laser facilities are expensive and precious. Easy-to-use software can support researchers in improving data quality and can support data analysis. The software *Condor* is a fast simulation tool specialized for FXI research and covers a wide range of use cases and functionalities. Practically anybody is able to use *Condor* because of its simple structure and because common hurdles such as limited cross-platform compatibility or demanding hardware requirements have been avoided by making key features available through a web application. We, the developers, encourage and support the integration of the code into other software that relies on simulated FXI data. Reusability of the source code is facilitated by the availability of a simple and flexible Python API and by the distribution of the code under the Simplified BSD license.

Beyond its relevance in research *Condor* may be a useful educational software tool. Students may gain understanding of the laws of X-ray diffraction by studying changes in the diffraction pattern while changing experimental parameters. Moreover, entire experimental data sets can be readily simulated by the students themselves. Students may be invited to pursue a reconstruction from simulated data.

In conclusion, *Condor* will enhance and stimulate collaborative activities in software development within the FXI community. Furthermore, the software will underpin efforts in FXI education, experiment planning, conducting of experiments, algorithm development and data validation.

## Figures and Tables

**Table 1:** Mass density, atomic composition and refractive index for a selection of optical media The material constants were taken from Bergh *et al.* (2008 ▸), Molla *et al.* (1991 ▸) and Dans *et al.* (1966 ▸), and the refractive index was calculated from these values with the relationship given by (10).

Material type	Density (g cm^−3^)	Atomic composition	Refractive index (λ = 1.240 nm)
Water	1.00	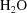	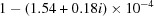
Protein	1.35	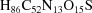	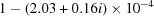
DNA	1.70	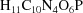	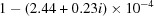
Lipid	1.00	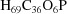	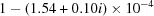
Cell	1.00	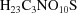	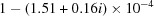
Poliovirus particle	1.34	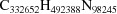 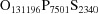